# A Novel Selection Marker for Efficient DNA Cloning and Recombineering in *E. coli*

**DOI:** 10.1371/journal.pone.0057075

**Published:** 2013-02-20

**Authors:** Chuan-Wei Jang, Terry Magnuson

**Affiliations:** 1 Department of Genetics, University of North Carolina, Chapel Hill, North Carolina, United States of America; 2 Carolina Center for Genome Sciences, University of North Carolina, Chapel Hill, North Carolina, United States of America; 3 Lineberger Comprehensive Cancer Center, University of North Carolina, Chapel Hill, North Carolina, United States of America; University of Massachusetts Medical School, United States of America

## Abstract

Production of recombinant DNA in bacterial cells is an essential technique in molecular biology. Plasmids are usually maintained in an *E. coli* host by antibiotic selection. However, there are only a few antibiotic-resistance markers available in common use. Here we report the adoption of a novel selection marker, *mfabI* (mutant *fabI*) for plasmid propagation in *E. coli*. *mfabI* expands the limited repertoire of selection markers and allows for more efficient molecular manipulation and plasmid propagation in *E. coli*. We show that *mfabI* is not only an efficient plasmid selection marker, but it also possesses unique activity that may facilitate molecular manipulation of unstable sequences. Furthermore, we have incorporated *mfabI* in the recombineering tool kit for generating mouse gene targeting vectors and demonstrate the advantage of using *mfabI*-containing recombineering vectors.

## Introduction

The two most commonly used selection agents in bacteria are ampicillin and kanamycin for the maintenance of plasmids bearing their corresponding resistance genes, beta-lactamase and neomycin phosphotransferase II, respectively [1], [2]. More antibiotic-resistance gene pairs have been discovered but are not commonly used for plasmid selection in bacteria. The chloramphenicol resistance gene is usually used for growth of BACs (bacterial artificial chromosomes) [3]. The tetracyclin resistance gene often serves as a negative selection marker in cloning vectors [4]. Moreover, chloramphenicol, tetracycline, and even kanamycin resistance genes have been frequently used in engineering of the bacterial genome to generate special host strains [5], [6], [7], and thus are precluded for conducting plasmid selection in engineered strains that already carry resistance to them. Resistance genes to blasticidin [8], hygromycin [9], and Zeocin [10] have also been developed into a variety of commercially available cloning vectors, but the high cost of these antibiotics generally precludes them from popular use. Therefore, the availability of a new selection marker would facilitate more efficient shuttling of recombinant DNA fragments between different vectors and allow for propagation in a broader range of special host strains.

Gene targeting by homologous recombination is an important method for mutating genes in the mouse. In spite of the progress of coordinated efforts to generate targeted mutant alleles for every annotated gene in the mouse genome [11], hypothesis-driven custom-made mutations at specific loci generated in individual laboratories remain an important source of novel discoveries. The recombineering technique is an efficient method commonly adopted by the research community to generate gene-targeting vectors, in which recombination between homologous regions in isolated DNA fragments, plasmids or BACs (bacterial artificial chromosomes) is catalyzed by bacteriophage enzymes expressed by engineered *E. coli* strains [12], [13]. A few antibiotic resistance markers are alternatively or simultaneously selected for at different steps to achieve efficient recovery of desired recombination products. However, the final gene-targeting vectors usually contain large fragments (>5 kb) of mammalian genomic sequence which are sometimes unstable or inefficiently replicated in the bacteria host, and therefore the recombination efficiency varies and desired vectors are sometimes difficult to obtain. We encountered a low efficiency problem associated with large complex sequences, and thus have improved the recombineering system by introducing a novel plasmid selection marker, *mfabI*.

Triclosan (IUPAC name: 5-chloro-2-[2,4-dichlorophenoxy]phenol) is a synthetic chemical which can inhibit bacterial growth in low concentrations and acts as a biocide in higher concentrations [14]. It exerts antibacterial activity by its inhibitory binding to a bacterial enzyme FabI (enoyl ACP reductase) [15], which is required for lipid synthesis in *E. coli* [16]. Spontaneous point mutations in FabI affecting the binding of triclosan to FabI allow the mutant bacteria to grow in the presence of triclosan. In the genomic library screens which identified FabI as the triclosan target, plasmids harboring wild-type or a mutant form (G93V) of the *fabI* gene were found to confer triclosan resistance to the transformed *E. coli* host [15]—suggesting its potential use as a plasmid selection marker. The wild-type *fabI* gene was further shown to be an effective plasmid selection marker in *E. coli* [17]. Here, we demonstrate that the G93V mutant form of *fabI* (*mfabI*) is an even more efficient selection marker with a unique growth suppression effect which may lead to stabilization of large or complex cloned sequences. We also developed *mfabI*-containing recombineering vectors to improve the recombineering tool kit for mouse gene-targeting construct production and demonstrate their advantage in practical use.

## Materials and Methods

### Plasmid construction

pF was constructed by PCR cloning and recombineering to replace the ampicillin resistance marker on pBluescript KS- with *fabI*. Primer sequences are listed in **Table 1**. *fabI* was amplified with Fab-F/Fab-R from genomic DNA of DH10B. The 5′ and 3′ homologous sequences were amplified from pBluescript KS- (pBS) (Stratagene) with Amp5-F/Amp5-R and Amp3-F/Amp3-R, respectively. The three fragments were PCR-spliced together with Amp5-F/Amp3-R, gel-purified, and then electroporated into heat-shocked SW106 [18] electrocompetent cells together with pBluescript KS-. Electroporated cells were selected on 1 µM triclosan plates. Correct clones were identified by restriction enzyme digestion of miniprep DNA and confirmed by sequencing with Amp5-F, Amp3-R, Fab-R1, and Fab-F1. The initial clones all contained mixtures of pBS and pF plasmids. pF DNA was further isolated by retransformation and miniprep screening.

**Table 1:** Primers used in plasmid construction.

Fab-F	AGACAATAACCCTGAGACCGAGCCGAATAGCTGTTGTGGT
Fab-R	GCTTAATCAGTGAGGCCGCCCATCTTTACCAACAGAACGAT
Amp5-F	GATAGGGTTGAGTGTTGTTCCAGTTTGGAAC
Amp5-R	CGGCTCGGTCTCAGGGTTATTGTCTCATGAGCGGATACA
Amp3-F	GATGGGCGGCCTCACTGATTAAGCATTGGTAACTGTCAGAC
Amp3-R	GTAACAGGATTAGCAGAGCGAGGTATG
Fab-R1	CAGCTGATCGCCAGGTGCAAAACCA
Fab-F1	GAGCTGAACTGGCATTCACCTACCAGA
Fab-G93V-R	GATCGCCAGGTGCAAAAACAATAGAGTGTACGAAACCGTCAAATTTCGG
Fab-G93V-F	GTACACTCTATTGTTTTTGCACCTGGCGATCAGCTGGA
Fab-BstE2-F	CGGTAAGCGCATTCTGGTAACCGGT
Fab-BssH2-R	GCTTTTGCCAGACCCATAACGTTGTAGTTC

pF2 was constructed by PCR mutagenesis. Using pF as template, two G93V mutation containing fragments were amplified by Fab-BstE2-F/Fab-G93V-R and Fab-G93V-F/Fab-BssH2-R, gel-purified, and then PCR-spliced together with Fab-BstE2-F/Fab-BssH2-R, resulting in the *mfabI* fragment. The *mfabI* fragment was cloned into pF to generate pF2 with *Bst*EII/*Bss*HII digestion. Transformed cells were selected with 50 µM triclosan. Correct clones were identified by restriction enzyme digestion of miniprep DNA and confirmed by sequencing with Fab-BstE2-F and Fab-BssH2-R.

pF-DTA was generated by recombineering with fragments from pDTA, which has the EF1a-DTA negative selection marker cloned into pBS backbone, cut by *Apa*LI deleting ampicillin resistance marker and pF by *Afl*III/*Dra*III containing *fabI*. pF2-DTA was generated by cloning *mfabI* into pF-DTA by *Fsp*I/*Apa*LI. pF2-DTA-Rosa26 was generated by inserting fragment from pRosa26-1 (Addgene plasmid 21714) [19] cut by *Eco*53kI/*Sal*I into the *Nru*I/*Sal*I sites in pF2-DTA.

### Bacterial transformation and growth

In transformation efficiency assays with DH10B cells, homemade retransformation grade chemical competent cells were used. Twenty-eight microliters of competent cells were heat-shock transformed with 2 µl of 100 ng/µl plasmid DNA, recovered at 32°C for 1.5 hr in 1 ml SOB, from which 50 µl of cells were plated on each plate. For assays with DH5α cells, 28 µl homemade cloning grade chemical competent cells, 2 µl of 1 ng/µl DNA, were used. For recombineering experiments, we follow the published protocols [12]; after 1.5-hr recovery, 200 µl from a 1 ml suspension was plated on each selection plate, incubated at 32°C overnight. Triclosan was purchase from VWR International (Cat. No. 80511-110, Triclosan antibiotic 1 gm, EMD Millipore), dissolved in DMSO, and alliquoted, stored in −20°C as 50 mM and 1 mM (1,000×) concentration stocks.

In the plating assays with clonal broth culture of pF- and pF2-transformants, colonies were picked into LB broth with 1 µM triclosan (untransformed DH10B cells in antibiotic-free LB broth), and cultured in 32°C shaker for 21 hrs. At the time of plating, amounts equivalent to 1.6×10^−3^ µl of OD600 = 1 culture were used for each sample on each plate.

In liquid culture assays, overnight clonal cultures were first spun down and resuspended in antibiotic-free LB broth to avoid any remnant antibiotic affecting the subsequent assay. O.D. equalized amounts of culture were inoculated into 15 ml LB broth in 50 ml conical tubes with different concentrations of triclosan, grown with constant 250-rpm shaking. At the end of culture, amounts equivalent to 2.1×10^−3^ µl of OD600 = 1 culture were used for each sample on each plate for the triclosan resistance retention assay.

In the retransformation assay in DH5α cells, 2.5, 5 and 10 ng of pF2, pF2-DTA-Rosa26 and pF2-DTA-Rosa26-Insert plasmids were used to transform 14 µl of homemade cloning grade chemical competent cells. After 1.5-hr recovery at 37°C, 150 µl from a 500 µl resuspension was spread onto 1 µM triclosan LB plates and grown for 24 hrs at 37 or 32°C before observation. Colonies were picked from plates incubated at 32°C and grown in LB broth with 1 µM triclosan at 37°C for 17 hrs before being measured and yielded for DNA extraction.

### Colony counting and statistics

Plates were scanned on a flatbed scanner with 300 dpi resolution, 8-bit, grey scale setting. Scanned images were first processed in Adobe Photoshop to enhance contrast by “Image>Adjustments>Levels” function, and then in Image J by first “Process>Binary>Make binary” and then “Process>Binary>Create watershed”. Colonies were counted in Image J by circling/selecting individual plates and using “Analyzed>Analyze Particles” function with following settings: Size = 6–200; Circularity = 0.75–1. Statistical analyses were done with Microsoft Office 2010 Excel by using “Data>Data Analysis>t-Test: Paired Two Sample for Means” function.

## Results

### Triclosan resistance conferred by *mfabI* plasmid

**Figure 1:**
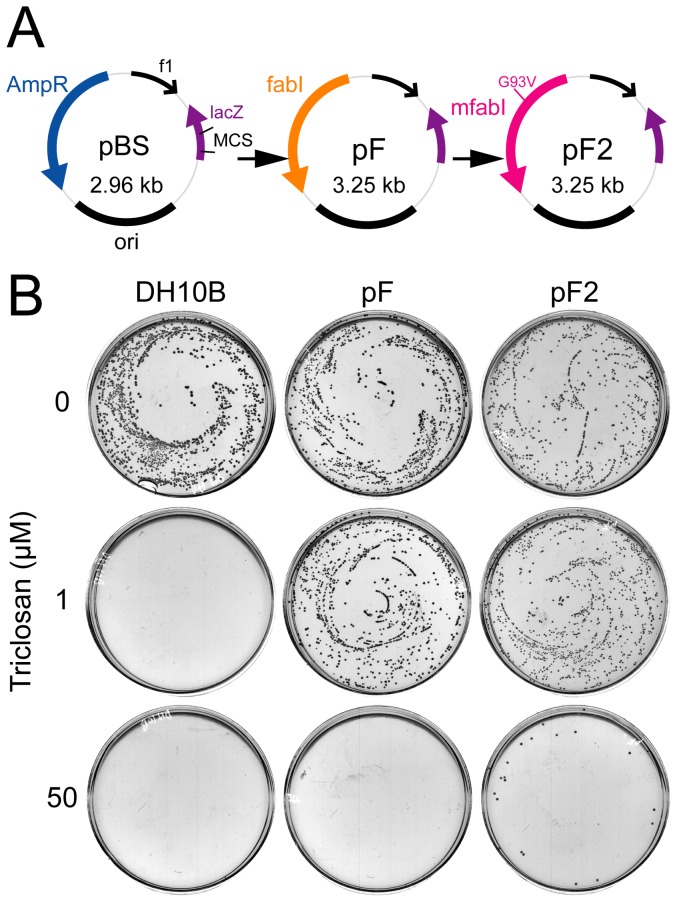
Strong triclosan resistance to host cells conferred by *mfabI* plasmid. (**A**) Schematic diagram of pF and pF2 vector consruction. Ampicillin resistance marker in pBluescript (pBS) was replaced by wild-type *fabI* gene to generate pF vector. The G93V mutation was introduced into *fabI* in pF, resulting in *mfabI*, to generate pF2 vector. (**B**) Colony formation of pF and pF2-transformed cells in triclosan selection. Equalized amounts of overnight broth cultures of pF, pF2 or untransformed DH10B cells were spread on LB agarose plates with 1 or 50 µM triclosan and incubated in 32°C for 24 hrs.

We cloned the *fabI* gene from the DH10B [20] bacterial genome using PCR methods. Vector pF was generated by replacing the ampicillin resistance gene in pBluescript (pBS) (Stratagene) with *fabI*. By PCR mutagenesis, the G93V point mutation was introduced into the *fabI* in pF, resulting in *mfabI*, to generate pF2 (Fig. 1A). The function of *fabI* and *mfabI*, and bacteriostatic activity of triclosan was reconfirmed by retransformation and plating assays. Triclosan efficiently inhibited the growth of untransformed bacteria, while *mfabI* marker conferred a stronger host resistance to triclosan than the wild-type *fabI* (Fig. 1B).

### Suppression of bacterial growth by *mfabI* plasmid

It was reported *fabI* in a plasmid can suppress the growth of the host cells in antibiotic-free medium, probably due to an imbalance in lipid metabolism in the cells caused by overproduction of the FabI enzyme [17]. Unexpectedly, *mfabI* exerts an even stronger growth suppression effect on host cells than that of *fabI* in the standard growth condition on solid support medium.

**Figure 2:**
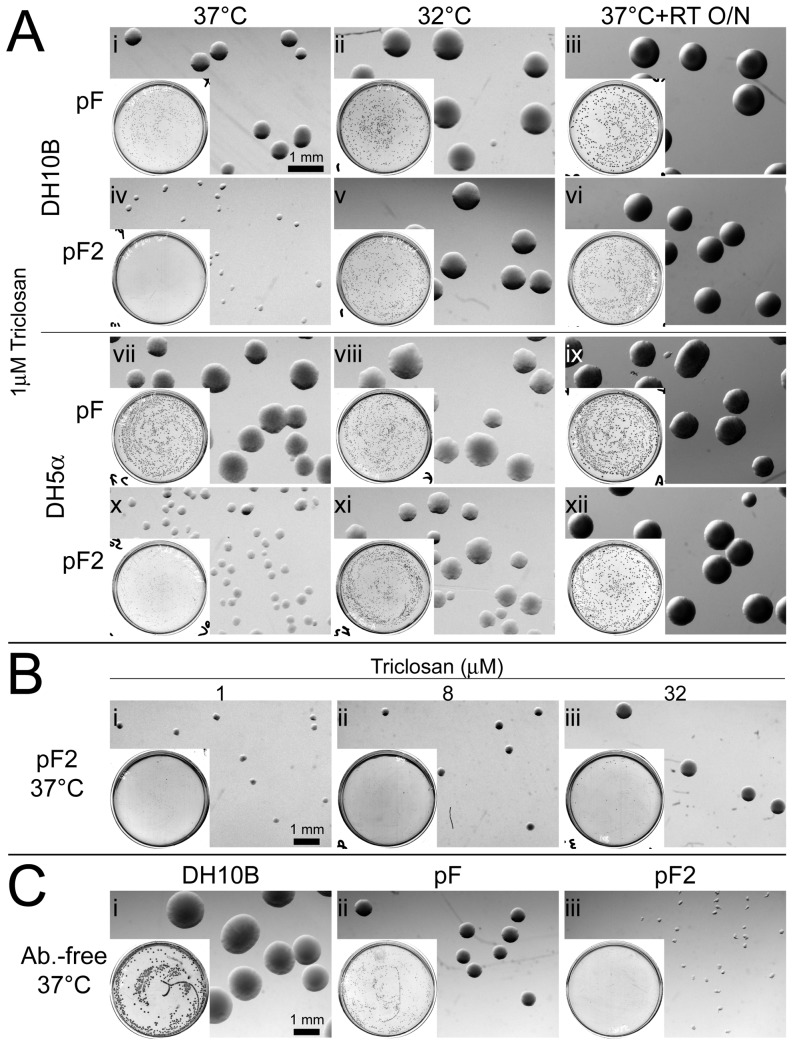
Impact of *mfabI* plasmid on host cell growth on solid support media. (**A**) Growth suppression of host cells by *mfabI* plasmid at standard growth temperature. Shown are colonies of DH10B (**i–vi**) or DH5α (**vii–xii**) cells grown on 1 µM triclosan plates; insert is the low-magnification view of the whole plates. Cells were transformed by pF (**i–iii & vii–ix**) or pF2 (**iv–vi & x–xii**) and grown in 37°C (**i, iv, vii & x**) or 32°C (**ii, v, viii & xi**) for 24 hrs. Plates incubated in 37°C were imaged again after additional 24-hr. incubation in room temperature (**iii, vi, ix & xii**). (**B**) Rescue of *mfabI* plasmid-induced growth suppression by higher concentration of triclosan. pF2-transformed DH10B cells were grown in different concentration of triclosan. Equal amounts of transformed cells were plated with 1 (**i**), 8 (**ii**), or 32 µM (**iii**) triclosan, and incubated in 37°C for 24 hrs. (**C**) Triclosan resistance-independent effect of *mfabI* on host cell growth. Clonal untransformed (**i**), pF- (**ii**), or pF2-transformed (**iii**) DH10B cells were plated on antibiotic-free plates and incubated in 37°C for 24 hrs. Scale bar is 1 mm.

When DH10B *E. coli* cells were transformed by pF or pF2 and grown in 37°C, the pF2 colonies were much smaller than pF colonies (Fig. 2A, compare i with iv). We also transformed another commonly used *E. coli* strain, DH5α[20], and observed a similar effect (Fig. 2A compare vii with x), indicating *mfabI*-induced growth suppression is not specific to DH10B cells but more general to most strains. However, when we used a lower growth temperature or a higher triclosan concentration during the construction of pF2 or manipulation of other *mfabI* plasmids, we did not observe a dramatic colony size reduction under these conditions. Therefore we examined the influence of temperature and triclosan on pF2 colony growth. Indeed, lower incubation temperature dramatically rescued the growth phenotype of pF2 colonies (Fig. 2A v and xi). In addition, the growth suppression by pF2 in 37°C was not permanent but could be subsequently relieved by shifting the cells into a lower temperature (Fig. 2A vi and xii). We also found elevated triclosan levels can boost the size of pF2 colonies, albeit reducing the total number of colonies (Fig. 2B).

However, with these results, it remained unclear whether the enhanced growth suppression effect of *mfabI* is solely due to its reduced sensitivity to triclosan—G93V mutation disrupts the interaction between FabI and triclosan [15], or the mutation has caused additional functional changes to the enzyme—since *fabI* also has a mild growth suppression effect on the host that can be reversed by triclosan [17]. To distinguish between these two possibilities, we plated clonal pF or pF2 cultures on antibiotic-free medium. While pF colonies showed reduced size compared to the wild-type cells, pF2 had a much stronger effect (Fig. 2C). These results indicate the mFabI enzyme has gained a hypermorphic or neomorphic function, in addition to its reduced sensitivity to triclosan, that contributes to the strong growth suppression effect.

**Figure 3:**
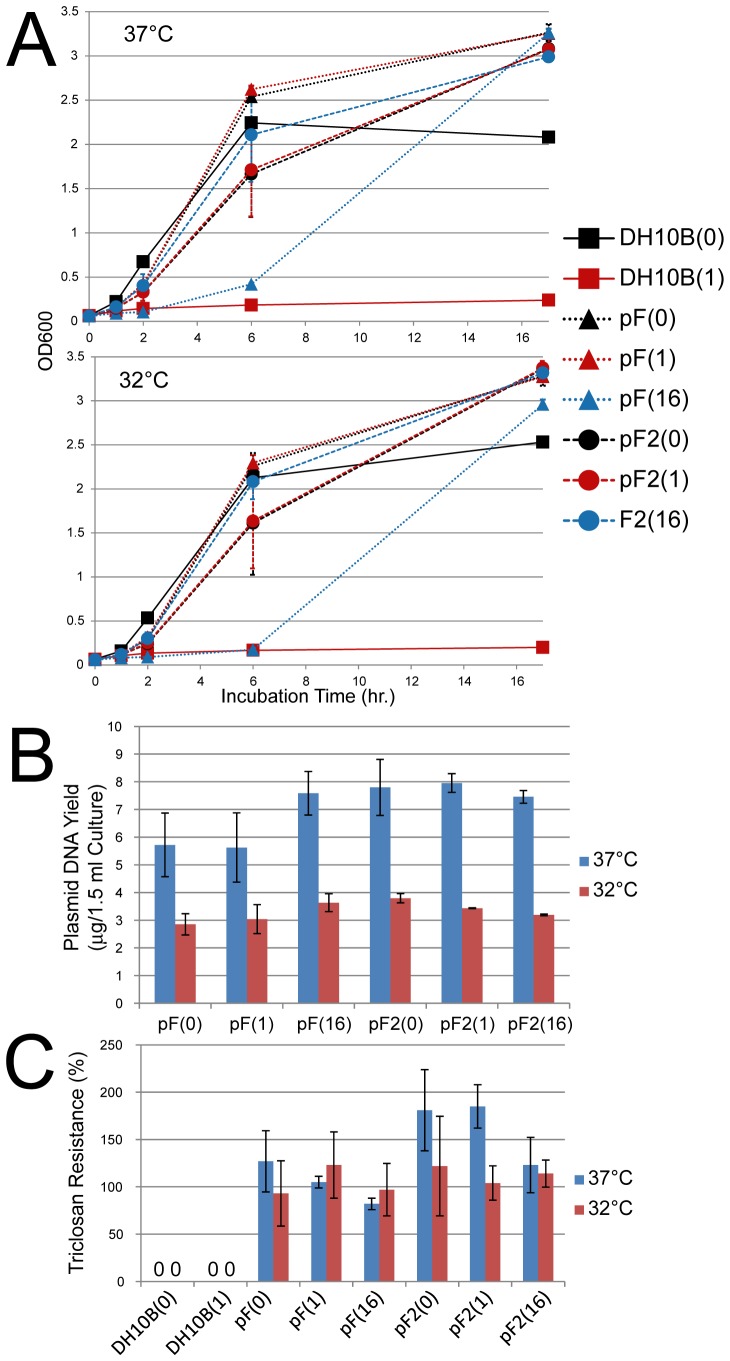
*mfabI* plasmid propagation in liquid culture. (**A**) Growth curves. Equalized overnight broth culture of untransformed DH10B, pF, or pF2-transformed clones (two for each; N = 2) were seeded in LB broth with different concentrations of triclosan (0, 1, or 16 µM; indicated by numbers in parentheses), and grown at 37 (left) or 32°C (right panel), with constant 250-rpm shaking. Samples were taken at different time points for OD measurement. (**B**) Plasmid DNA yields of pF and pF2 with different growth conditions. DNA was extracted from the cultures in the growth curve experiment shown in (A) at the end point (17 hr.). (**C**) Plasmid retention (triclosan resistance) after different culture conditions. Equalized amounts of cells from the end point cultures in the growth curve experiments were plated with or without 1 µM triclosan. Triclosan resistance was calculated by dividing the number of colonies formed on the triclosan plate by that on the antibiotic-free plate for individual cultures. pF and pF2 clones tend to form more colonies when plated with triclosan, resulting in a counterintuitive >100% triclosan resistance; 16 µM triclosan-cultured cells might have developed higher triclosan dependency, and thus were less enhanced by 1 µM triclosan on colony formation. Error bars show standard deviations.

We also analyzed the growth characteristics of pF and pF2 clones in liquid culture. The growth suppression effect of *mfabI* and its rescue by triclosan was relatively mild and only evident in early growth phase (Fig. 3A). Lower incubation temperature had a marginal effect on the bacterial growth (Fig. 3A). Plasmid yields were not significantly affected by triclosan concentration, but generally higher at 37°C (Fig. 3B). Plasmids were well maintained at the end of culture in all conditions (Fig. 3C).

### Efficient transformation of *E. coli* by *mfabI* plasmid

**Figure 4:**
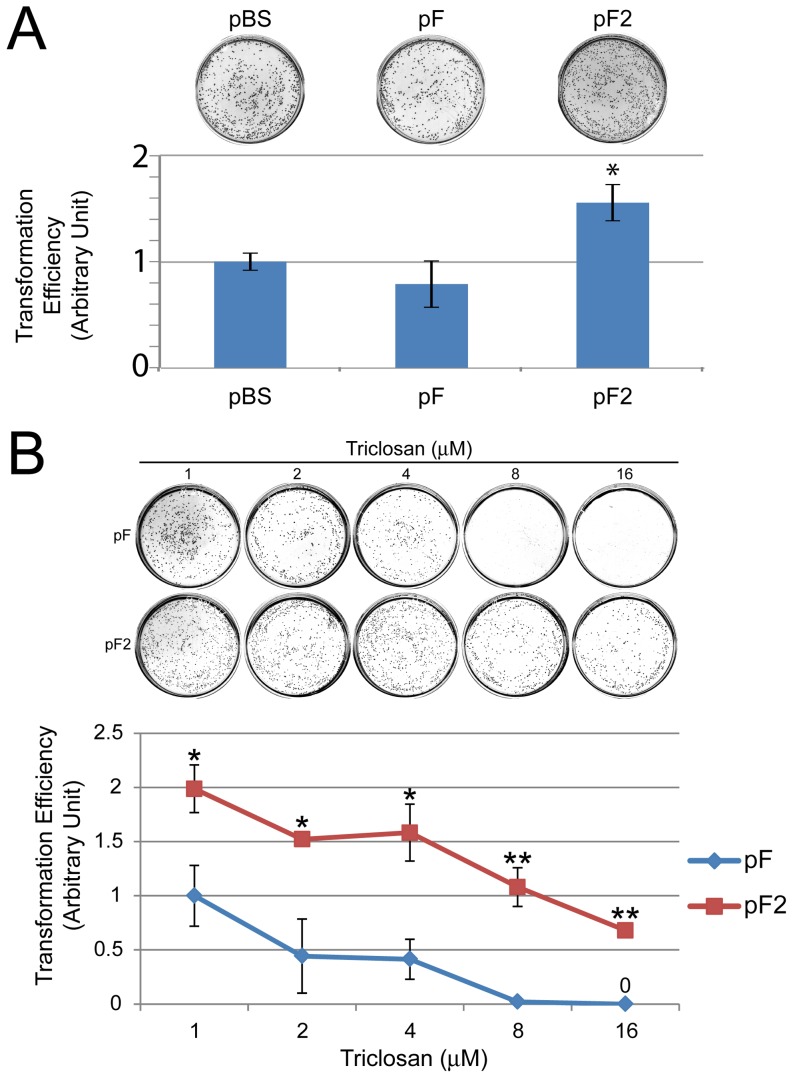
Superior transformation efficiency and robust triclosan resistance of *mfabI*. (**A**) pF2 has a higher transformation efficiency than that of pBS and pF. Equal amounts of plasmid DNA were used to transform DH10B cells, which were then plated on 100 µg/ml ampicillin (for pBS-transformed cells) or 1 µM triclosan plate (for pF and pF2-transformed cells). Transformation efficiency of each plasmid was normalized to that of pBS. Data were collected from three independent replicates (N = 3). pF2 had significantly higher transformation efficiency than that of pBS. (**B**) More robust triclosan resistance with *mfabI* plasmid than that with *fabI* plasmid. DH10B cells were transformed by pF or pF2 and plated with different concentrations of triclosan. Transformation efficiency of each plasmid at each concentration was normalized to that of pF2 at 1 µM triclosan. The graph is generated with data from three independent replicates (N = 3). pF2 shows significantly higher transformation efficiency than that of pF at each concentration tested. *: p<0.05 **: p<0.01 in two-tailed, paired t-test. Error bars show standard deviations.

To evaluate *mfabI* function as a plasmid selection marker, we assessed the transformation efficiency of pF2. When analyzed, pF2 showed transformation efficiency 50% higher than that of pBS (Fig. 4A). We then compared pF2 and pF for their triclosan resistance (Fig. 4B). The transformation efficiency of both pF and pF2 inversely correlates with the triclosan level. This may reflect the increasing bactericidal activity with increasing concentration of triclosan. pF2 consistently showed higher transformation efficiency than pF at any triclosan level examined. Taken together, *mfabI* appears to be a superior plasmid selection maker with high transformation efficiency and robust triclosan resistance.

### Efficient recombineering with *mfabI* vectors

**Figure 5:**
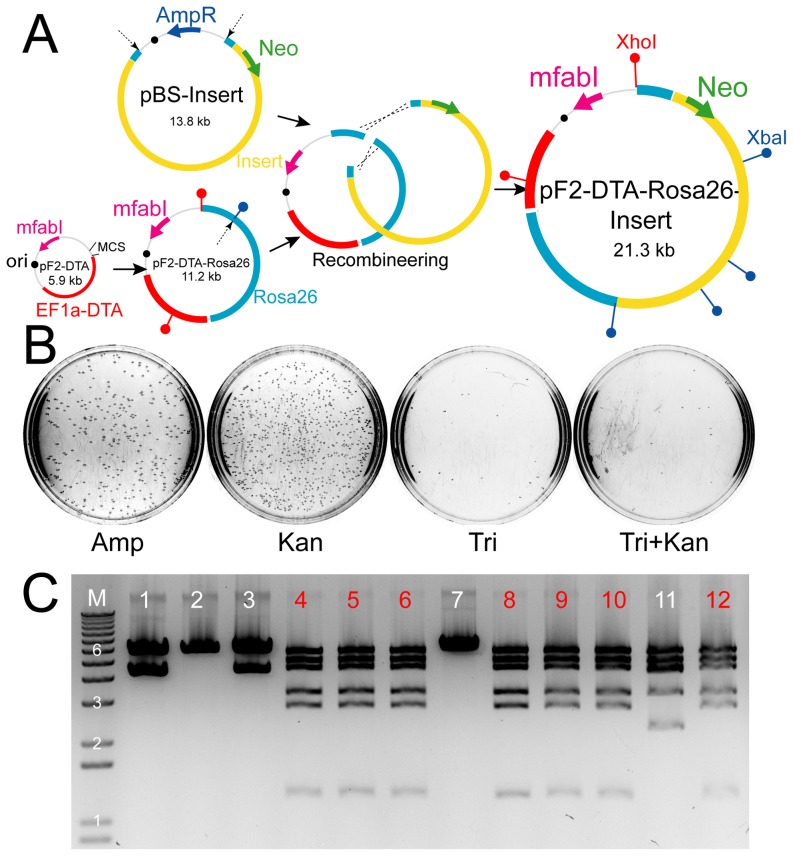
Recombineering with *mfabI* plasmids. (**A**) Schematic of the recombineering scheme. *mfabI* marker was incorporated in the retrieval plasmid containing the DTA marker. Neo selection marker is in the insert fragment. Through, homologous recombination, the final targeting vector plasmid carries both *mfabI* and Neo markers. Dashed arrows indicate restriction enzyme site locations on the plasmids used for linearization or isolation of fragments for recombineering. Black circles denote the replication origin on plasmids. The positions of XhoI and XbaI diagnostic sites are indicated by red and blue lollipop signs on the maps. (**B**) Equal amounts of electroporated heat-shocked SW106 cells were plated on ampicillin (Amp), kanamycin (Kan), triclosan (Tri), or triclosan/kanamycin double (Tri+Kan) selection plates and incubated in 32°C for 24 hrs. (**C**) Restriction enzyme (XhoI/XbaI)-digested miniprep plasmid DNA from clones picked from the double selection plate shown in (B). M is DNA molecular weight marker lane (Invitrogen 1 kb plus DNA ladder); numbers denote kb bands. Clones numbered in red had the correct digestion pattern.

As our original goal in developing a new plasmid selection marker was to solve recombineering problems, we incorporated *mfabI* into vectors for recombineering to obtain our desired gene targeting vector. *mfabI* was used to replace the ampicillin resistance gene in pDTA to create pF2-DTA (Fig. 5A). After correct recombination, the resulting targeting vector should carry both *mfabI* and Neo, and confer host resistance to both triclosan and kanamycin. Cells were plated with different selective agents to demonstrate improved efficiency with *mfabI* vectors (Fig. 5B). With ampicillin selection, colonies represent background contamination of the pBS-Insert plasmid. Usually this is not a serious problem, but in our case the restriction enzyme digested insert fragment was large and migrated close to the incompletely digested plasmid in gel electrophoresis, resulting in substantially high number of ampicillin resistant colonies. In the original recombineering scheme, this would interfere with identification of recombined clones, in which pDTA-Rosa26 would also carry ampicillin resistance gene and the double selection with kanamycin and ampicillin would still select for undigested pBS-Insert. When triclosan was applied in the double selection in our improved system, we recovered very few colonies, indicating all the ampicillin resistance background colonies were eliminated. We screened twelve colonies and identified seven correctly recombined clones (Fig. 5C). The incorrect clones showed restriction enzyme digestion patterns different from that of either pBS-Insert or pF2-DTA-Rosa26, and presumably resulted from aberrantly recombined clones missing unknown regions of the cloned sequences. The insert we constructed contained complex sequence repeats which may have contributed to the high aberrant recombination rates. We also attempted recombineering with pF-DTA-Rosa26, which has the same sequence composition as pF2-DTA-Rosa26 but with the wild-type *fabI* as the selection marker, but had less successful results (data not shown). Only three out of ten clones screened had the correctly recombined product; the remaining harbored aberrant recombinations. In addition, the yield of DNA from the correct clones was two thirds less than that with *mfabI* vectors.

### Growth advantage conferred by larger *mfabI* plasmids

**Figure 6:**
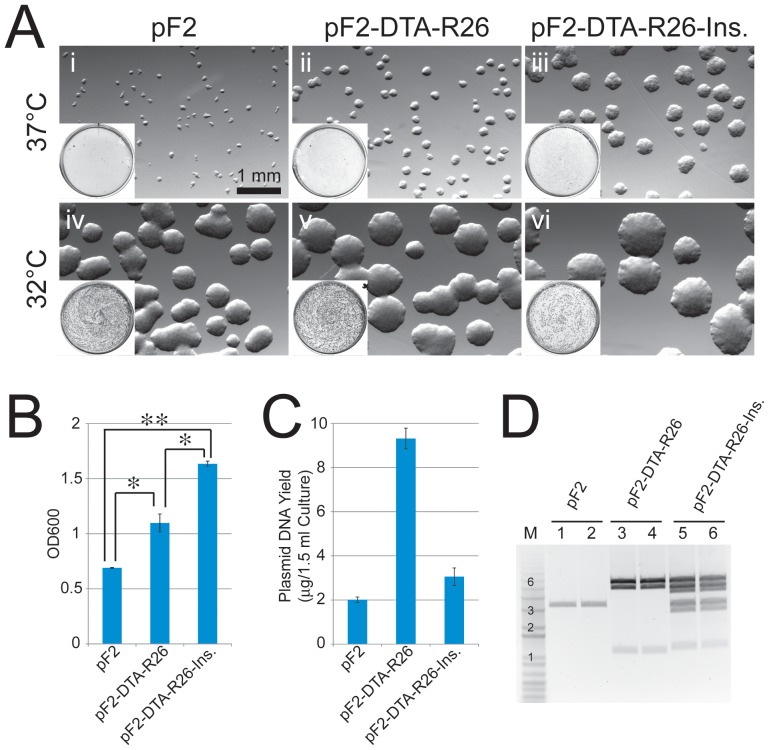
Cells transformed with larger *mfabI* plasmids have a growth advantage. (**A**) Larger colonies were formed by cells transformed with larger *mfabI* plasmids on plates. Shown are colonies of DH5α cells transformed by pF2 (**i & iv**), pF2-DTA-Rosa26 (**ii & v**), or pF2-DTA-Rosa26-Insert (**iii & vi**) and grown at 37°C (**i, ii & iii**) or 32°C (**iv, v & vi**) on 1 µM triclosan LB plates; insert in each panel is the low-magnification view of the whole plate. (**B**) Higher cell densities obtained in overnight cultures of larger *mfabI* plasmid transformants. Two colonies per group were picked from 32°C-grown plates and grew in 1 µM triclosan LB broth at 37°C for 17 hrs before O.D. measurements. *: p<0.05 **: p<0.01 in one-tailed, unpaired t-test (N = 2). Error bars show standard deviations. (**C**) Plasmid DNA yields from overnight cultures. Plasmid DNA was extracted from cultures used in (B). Error bars show standard deviations (N = 2). (**D**) Restriction enzyme (XhoI/XbaI)-digested plasmid DNA used in (C). Molar equalized amounts of DNA were digested. All clones showed expected correct digestion patterns (refer to maps in Fig. 5A). M is DNA molecular weight marker lane (Invitrogen 1 kb plus DNA ladder); numbers denote kb bands.

*mfabI* appeared to promote preservation of larger constructs, since we had a higher efficiency in obtaining the gene targeting vector with *mfabI* constructs in our recombineering experiments. To further test the large plasmid stabilization effect of *mfabI*, we transformed cells with pF2 (3.25 kb), pF2-DTA-Rosa26 (11.2 kb), or pF2-DTA-Rosa26-Insert (21.3 kb) and observed their growth phenotypes. On selection plates, larger colonies were formed with larger constructs, although the differences were reduced at a lower growth temperature (Fig. 6A). In overnight broth culture, higher cell densities were achieved with larger constructs (Fig. 6B); although the DNA yields do not always correlate to cell densities (Fig. 6C). The integrity of larger plasmids was well preserved in the cultures as shown in restriction enzyme digestions (Fig. 6D). In summary, cells transformed with larger *mfabI* plasmids have a growth advantage on solid medium and in liquid culture. The effect may lead to stabilization of larger plasmids grown in *E. coli* cells.

## Discussion

Through detailed characterization, we have demonstrated that *mfabI* is an efficient plasmid selection marker for molecular cloning applications in *E. coli*. The introduction of a new selection marker will lead to more efficient molecular manipulations and may facilitate more complicated experimental designs. As demonstrated in our recombineering experiment, the employment of *mfabI*-incorporated recombineering vectors solved the low efficiency problem we encountered associated with high background contamination of undigested ampicillin plasmid, which is significantly better than the original recombineering strategy using only two plasmid selection markers. A practical and valuable advantage with *mfabI* marker is the low cost of its selective agent triclosan, making it easily applicable in any laboratory.

It is not surprisingly that the *mfabI* marker shows stronger resistance to triclosan than *fabI*, since the G93V point mutation was found in the triclosan-resistant *E. coli* mutant genome. However the stronger growth suppression effect of *mfabI* plasmid on the host was unexpected. Even when grown in the absence of triclosan, the difference between *mfabI* and *fabI*-transformed colonies was dramatic, suggesting the G93V mutation changes the property of the FabI enzyme beyond merely interrupting its interaction with triclosan. The boost of colony size by higher concentrations of triclosan indicated the G93V mutation may enhance enzymatic activity. The mutation may stabilize mFabI protein or render it resistant to cellular regulation, and the resulting elevated or uncontrolled enzymatic activity affects bacterial growth more than wild-type FabI. Lower temperature may reduce the mFabI enzymatic activity or ease cellular stress, and therefore alleviate the growth phenotype. In practical applications, 32°C incubation should be used for positive selection. The strong growth suppression effect in 37°C suggests *mfabI*'s potential use as a negative selection marker.

**Figure 7:**
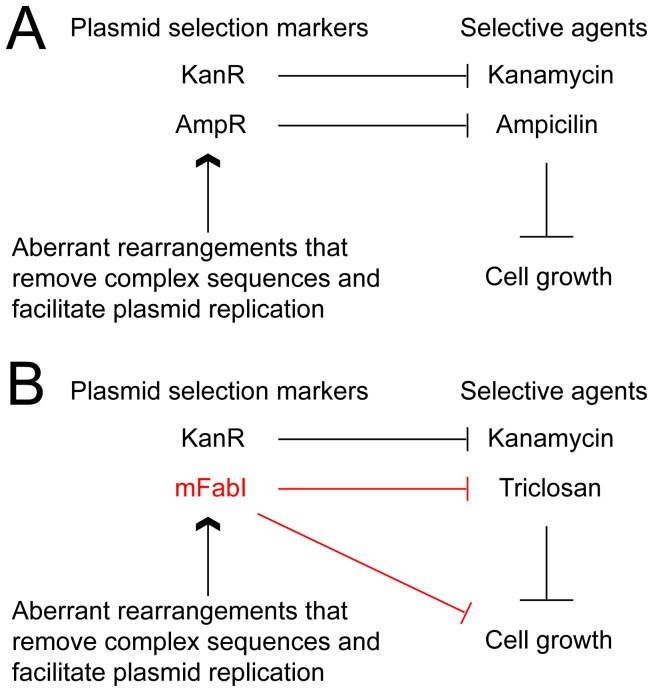
Suppression of undesired sequence rearrangement events with *mfabI* marker in the recombineering system. (**A**) In the original recombineering system, undesired sequence rearrangement events are favored in the selection because they promote cell growth. (**B**) In the recombineering system with *mfabI* marker, undesired sequence rearrangements are suppressed, since increased mFabI activity can suppress cell growth.

In the generation of our gene-targeting vector via recombineering, *mfabI* appears to help retain large and complex inserts. Furthermore, cells transformed with larger *mfabI* plasmids with more complex sequences grow better than that with a smaller *mfabI* vector in both solid and liquid media. Based on these results, we propose a model of strong growth suppression activity of *mfabI* leading to suppression of unwanted aberrant sequence rearrangement events, which happens frequently in cloning of large or complex sequences. In the original recombineering scheme (Fig. 7A), both ampicillin and kanamycin resistance markers promote host cell growth under selection. When the desired plasmid contains complex sequences that do not replicate efficiently, the selection pressure favors loss of sequences, thus producing more resistance gene products. In the *mFabI*-incorporated system (Fig. 7B), although mFabI product is required for host cell survival in triclosan selection, it also suppresses host cell growth. Aberrant rearrangement events that remove unstable sequence and thus enhance plasmid replication/mFabI production would have a negative impact on cell growth, and thus are disfavored in the selection, thereby becoming less frequent. This mutation suppression effect associated with *mfabI* should also be applicable in cloning any large complex sequences by other methods.
